# Hybrid manganese dioxide-bovine serum albumin nanostructure incorporated with doxorubicin and IR780 for enhanced breast cancer chemo-photothermal therapy

**DOI:** 10.1080/10717544.2019.1693706

**Published:** 2019-11-23

**Authors:** Xiao Yuan, Yanlong Yin, Wang Zan, Xiyang Sun, Qian Yang

**Affiliations:** a School of Pharmacy, Sichuan Province College Key Laboratory of Structure-Specific Small Molecule Drugs, Chengdu Medical College, Chengdu, China;; b Tongren Hospital, Shanghai Jiao Tong University School of Medicine, Shanghai, China

**Keywords:** Stimuli-responsive, hybrid nanoparticles, chemotherapy, combinational therapy

## Abstract

The therapeutic outcome of chemotherapy is limited, although it is still the preferent strategy for cancer therapy. By regulation of tumor microenvironment and introduction of another therapeutic manner for combination therapy can enhance the anticancer activity of chemotherapeutics. Herein, we have constructed a hybrid nanostructure which composed of manganese dioxide (MnO_2_) and doxorubicin (DOX) as well as IR780 by stabilizing with BSA (BMDI) in one-pot procedure to alleviate tumor hypoxia and enhance tumor growth inhibition. The MnO_2_ can react with H_2_O_2_ to generate oxygen, and additionally react with GSH to realize tumor microenvironment responsive drug controlled release. And the release Mn ions further enhanced the magnetic resonance signal which made the BMDI a promising contrast agent for MRI. Moreover, the introduction of MnO_2_ has enhanced the anticancer activity of DOX *in vitro* and *in vivo*, and efficiently suppressed the tumor growth. By further introducing with photothermal therapy (PTT), the tumor growth was almost inhibited. It demonstrated that the BMDI hybrid nanostructure has great potential in tumor growth inhibition as therapeutics carrier.

## 1. Introduction

Chemotherapy is the dominant strategy for treating various types of cancer (Dagogo-Jack & Shaw, 2018). It can directly inhibit the growth of cancer by inducing the apoptosis of cancer cells which makes it have unique advantage in cancer therapy (Zuccala, 2016). However, because of the heterogeneity of the tumor as well as the promotion of tumor microenvironment (TME), the therapeutic activity of chemotherapeutics has been dampened (Mantovani et al., 2017; Liu et al., 2018a). And many recent findings have indicated that the introduction of TME regulation and the combination of multidrug treatments or the targeted drug delivery systems can reverse the suppression of the activity of chemotherapeutics (Kemp et al., 2016; Peng et al., 2019a).

Tumor acidosis and hypoxia are the two ubiquitous features of the TME (Harris, 2002; Gatenby & Gillies, 2004). Among them, hypoxia has been proved to have negative effects on the therapeutic outcome of chemotherapy by changing the metabolic environment of cancer cells (Martin et al., 2016). It provides a harsher intratumoral environment, thus, lead to cancer cells metastasis and accelerate malignant progression (Robertson-Tessi et al., 2015; Carnero & Lleonart, 2016). Moreover, it may favor the formation of drug resistance in cancer cells, which has directly attenuated the efficacy of chemotherapy, and ultimately resulted in worse survival and recurrence (Wu et al., 2009). Amount of research work have proved that the alleviation of hypoxia indeed can reduce the expression of HIF-alpha, and favor the enhancement of the therapeutic outcome of some chemotherapeutics (Prasad et al., 2014; Rey et al., 2017; Lin et al., 2018). Therefore, regulation of the hypoxia in tumor microenvironment will be a potential strategy to enhance the therapeutic outcome of chemotherapy.

Kinds strategies have been developed to regulate the hypoxic environment, such as using metformin to reduce the consumption of oxygen by regulating the metabolic behavior of cancer cell or injecting perfluorinated compound (PFC) to directly delivering oxygen to the tumor tissues, etc. (Pernicova & Korbonits, 2014; Li et al., 2017; Zhou et al., 2019). However, these strategies still need to be improved, e.g. metformin cannot provide extra oxygen while PFC cannot achieve activated oxygen release. Therefore, it calls for substitute strategies. The nano-biological effects of some nanomaterials, e.g. manganese oxides provide an alternative choice for hypoxia regulation. Manganese oxides can react with hydrogen peroxides or GSH to generate oxygen. Some previous studies have been reported that, the administration of manganese dioxide could be employed to effectively alleviate the hypoxia of tumor *in vivo* (Pan et al., 2017; Peng et al., 2017b). Moreover, manganese oxides can also be introduced to construct H_2_O_2_-responsive and GSH-responsive nanostructures, which can be a promising stimuli-responsive contrast agent for tumor imaging, and also promote the achieving of activated drug release, favoring the improvement of chemotherapy (Chen et al., 2016; Feng et al., 2018; Zhou et al., 2018). And the MnO_2_ can be formed *in situ* via the biomineralization process with proteins, such as bovine serum albumin (BSA). It provides targeting-delivery strategy for MnO_2_ delivery. MnO_2_ can be served as stimuli-responsive agent for hypoxia alleviation.

Besides, numerous studies have been demonstrated that the introduction of phototherapy may further strengthen the tumor-growth inhibition by regulating the expression of various proteins as well as inducing cancer cell apoptosis directly, and in most cases, the phototherapy, especially the photothermal therapy, results in necrosis (Abbas et al., 2017). Among the numerous agents which have been invented to act as mediums for phototherapy, such as gold nanostructures, carbon-based nanostructures, or metal oxides, photosensitizers, especially the near infrared photosensitizers, have great potential in phototherapy (Yang et al., 2018; Huang et al., 2019; Liu et al., 2019). They not only can be served as the agents for photothermal therapy and photodynamic therapy, but also can be acted as probes or contrast agents for fluorescence imaging and photoacoustic imaging due to their inherent behaviors after light agitation (Gai et al., 2018; Liu et al., 2018b; Yang et al., 2019a). Various chemotherapeutics-induced chemotherapy has been incorporated with phototherapy to enhance the final therapeutic outcome and gained some improving results (Hu et al., 2018; Peng et al., 2019b). However, more suitable strategies still need to be developed to integrate the advantages of hypoxia regulation and phototherapy to enhance the tumor growth inhibition of chemotherapy synchronously.

Therefore, in this study, a hybrid nanostructure incorporating with manganese dioxide, photosensitizer and chemotherapeutics was initially constructed, and the combination of alleviation of hypoxia inside tumor and the PTT to enhance the therapeutic outcome of chemotherapy were further investigated. BSA was chosen as the carrier for its versatile loading capacities. IR780 was used as the photosensitizer which has high efficacy in photothermal conversion in our previous studies. Manganese dioxide was grown in situ and stabilized by BSA. And DOX was chosen as the model anticancer drug. All the components were incorporated by BSA in one-pot procedure. The followed stimuli responsive performance for drug release and MR imaging of the obtained hybrid nanostructure were studied in details. The *in vitro* and *in vivo* antitumor studies demonstrate that the responsive drug release and oxygen generation, combined with the laser irradiated hyperthermia by hybrid nanostructure, dramatically benefit for the tumor-growth inhibition, whereby alleviating the hypoxia of tumor region during photothermal-chemotherapy.

## 2. Materials and methods

### 2.1. Materials

MnCl_2_ .4H_2_O_,_ NaOH and H_2_O_2_ were purchased from Kemiou chemical company (Tianjin, China). IR780 iodide, DAPI, MTT Formazan powder and Live/Dead cell double staining kit were all obtained from Sigma-Aldrich Company (Saint Louis, USA). Doxorubicin (DOX, Mw: 543.52 g/mol) was obtained from Meilun Biotechnology, Co. Ltd (Dalian, China). Fetal bovine serum, RPMI-1640 medium, penicillin and streptomycin were all purchased from HyClone (GE, USA).

### 2.2. Synthesis and characterization of BSA-MnO_2_-DOX&IR780 nanostructure (BMDI)

#### 2.2.1. Procedure of BSA-MnO_2_-DOX&IR780 synthesis

BSA-MnO_2_-DOX&IR780 nanostructure (BMDI) were obtained by the in-situ precipitation of MnO2 on BSA in the presence of DOX and IR780. Topically, DOX and IR780 (with ratio of 6:1) were mixed in 6 mL BSA solution (10 mg/ml), and subsequently 0.03 mL MnCl2 solution (0.1 M) was added in drop under vigorous stirring. Consequently, NaOH solution (1.0 M) was added to adjust to ∼ pH11 to reduce the Mn^2+^. After 2 h reaction at 30 °C to introduce the nanostructure growth, the products were further dialyzed for 10 h to purify the obtained BMDI structures.

As the control, BSA-DOX&IR780 nanostructure (BDI) were obtained with the same procedure without the MnCl_2_ added. For the drug-loading investigation, the BI and BD were formed by simply mixing of DOX solutions or IR780 with BSA and then dialyzed over night at room temperature, which might contribute to adsorption of the positively charged DOX or IR780 molecules with the BSA protein.

#### 2.2.2. Morphology characterizations

The morphology of BMDI, and GSH-reduced BMDI were observed by the field-emission high resolution transmission electron microscope (HRTEM, Tecnai G2 F20 S-TWIN). The presence of Mn in the nanostructure was further confirmed by Spectroscopy (EDX).

Size distribution and zeta-potential for each sample were detected by the Zetasizer Nano ZS90 (Malvern) at room temperature. All results are presented as the mean of three repeated trials. The optical absorptions of BMDI with or without GSH-treated were measured by UV/Vis-spectrometer (UV-2600, Shimadzu). DOX emission spectra and IR780 emission spectra of BMDI with or without GSH-treated were monitored by fluorescence spectrophotometer (RF-6000, Shimadzu) at the excitation wavelength of 480 nm and 740 nm, respectively.

### 2.3. *In vitro* responsive MR imaging

The responsive MRI signals of BMDI dispersion were measured under different environment by the MR Instruments (BioSpec70/20USR, Bruke). The field strength of magnet was 7 Tesla. To test the contrast ability, the BMDI were diluted in PBS buffer with different concentrations, and blank PBS buffer was used as control. The effect of pH validation and GSH concentration on T1 signal of BMDI were studied following our previous protocol.

### 2.4. Oxygen generation of BMDI

The generation of oxygen via BMDI catalysis was detected by the Oxygen Meters (JPB-608, Shanghai Instrument Factory) based on our previous study (Peng et al., 2017a). Briefly, H_2_O_2_ solution (30%, w/v) was injected into a sealed glass flask (with dissolved oxygen probe inside) containing 100 mL of deoxygenated water until the final concentration of H_2_O_2_ reached 0.1 mM. When the recorded value of oxygen detector became stable, BMDI dispersion was injected into H_2_O_2_ solution in drop. The values of oxygen generated by BMDI were discontinuously monitored by the probe at predetermined time points, while gentle shaking was performed during the detection.

### 2.5. Drug loading and *in vitro* responsive DOX release profile

The amount of DOX in the obtained BMDI or BDI nanostructure was detected by UV-Vis spectrometer at 485 nm, while 790 nm was used for IR780 detection. All the results were the average of three replicates.

The drug loading capacity was calculated by the formula as follow:

Drug loading (DL)=(Weight of drug/Total weight of nanostructure)×100%

*In vitro* responsive drug release profiles were investigated at various environments. BMDI dispersion were placed separately in dialysis bags and immersed in different buffers, including PBS (pH 7.4) and PBS (pH 6.5), PBS (pH 6.5) with 100 μM H_2_O_2_, and PBS (pH 6.5) with 100 μM H_2_O_2_ and 10 mM GSH. At predetermined time points, the buffers containing released drug were collected and all replaced by fresh medium. The concentrations of DOX in the release medium were determined by UV-Vis spectrometer.

The responsive release behavior of BMDI was further studied by changing the release mediums. BMDI dispersion was primarily placed in PBS buffer (pH 7.4) for 24 h, and the alternate release medium PBS buffer (pH 6.5) containing 100 μM H_2_O_2_ and 10 mM GSH was substituted. The relationship of DOX release versus time was obtained.

### 2.6. *In vitro* cellular assay

#### 2.6.1. Cell lines

MCF-7 breast cell lines were purchased from American Type Culture Collection (Rockville, MD), which was cultured in RPMI 1640 medium containing10% of FBS, and 1% of penicillin/streptomycin, respectively. The culture condition was maintained at 37 °C, and with 5% CO_2_ atmosphere.

#### 2.6.2. For cell uptake assay

MCF-7 breast cancer cells in 6-well plates were pre-cultured overnight. After exposed to BMDI, BDI and free DOX dispersion for 4 h, cells were labeled with DAPI followed by three times washing, and then observed consequently by confocal microscopy (Zeiss OBSERVER D1/AX10 cam HRC).

#### 2.6.3. The cytotoxicity of BMDI was investigated by MTT assay

The MCF-7 cells were pre-cultured in 96-well plates with density of 5 × 10^3^ cells/well. After different concentrations of free DOX/IR780 mixture, BM, BI, BD, BDI, and BMDI treated, the MTT assay was conducted for the determination of cell survival 24 h after co-culture. The data reported represent means ± SD (*n* = 6).

#### 2.6.4. For photothermal therapy

MCF-7 cells were seeded and mixed with free IR780 or BMDI dispersion. After 4 h, the 12-well plates were exposed to the 808 nm laser for 5 min (1.5 W/cm^2)^. Then, cells were transferred into fresh medium and further incubated for 4 h before the calcein AM/PI co-staining performing (Live/Dead Cell Double Staining Kit). The living/dead cells images were captured by fluorescence microscope (Olympus IX2-ILL100).

#### 2.6.5. Cell survival assay was conducted for different BSA-based formulations

The MCF-7 cells were seeded in 96-well plates (5 × 10^3^ cells/well) and cultured for 24 h. After 4 hours’ co-incubation with different concentration of free DOX/IR780 mixture, BI, BDI, and BMDI, the fresh medium was replaced for each well. And then, some wells were irradiated by 808 nm laser for 5 min. MTT assay was carried out for the determination of cell survival another 20 h after treatment. The data reported represent means ± SD (*n* = 3).

### 2.7. *In vivo* studies

#### 2.7.1. Animals

Balb/c nude mice (6-8 weeks old) were purchased from Vital Laboratory Animal Center and kept under SPF condition with free access of food and water. Additionally, the procedure for all animal experiments were followed by the Guiding Principles for the Care and Use of Laboratory Animals of Chengdu Medical College, China

#### 2.7.2. Tumor targeting efficiency study

IR780, as an NIR fluorescent dye, was utilized for the visualization the BMDI enrichment in tumor model by fluorescence imaging. Before the *in vivo* targeting investigation, BMDI dispersion with different concentrations were applied to measure the fluorescence intensity *in vitro* (the excitation wavelength and the emission wavelength were 740 nm and 790 nm, respectively) by IVIS Lumina XR system (PerkinElmer)

MCF-7 tumor cells were subcutaneously inoculated on mice for tumor model established. After the tumor volume growing up to about 200 mm^3^, mice were administrated with free IR780 and BMDI intravenously (at an equivalent IR780 concentration of 1.5 mg/kg, based on our previous study). Fluorescence images were captured by IVIS Lumina XR system at predetermined time points of 1, 2, 8, 24 and 32 h. After 32 h, all mice were euthanatized and their organs were applied for *ex vivo* imaging.

#### 2.7.3. Anticancer performance *in vivo*

Antitumor activity of BMDI was evaluated on MCF-7 tumor burden mice. After the average tumor volume reaching about 160 mm^3^, mice were divided randomly into five groups (*n* = 5): (1) i.v. injected with saline; (2) i.v. injected with free DOX; (3) i.v. injected with BDI; (4) i.v. injected with BMDI; (5) i.v. injected with BMDI and irradiated by NIR laser for 5 min 24 h after first injection (808 nm 1.5 W/cm^2^). The total doses of DOX was kept at 9.0 mg/kg for each group, and each treatment was administered daily for 3 days. For the BMDI plus 808 nm laser treated group, the temperature validation of tumor region was recorded by infrared imaging camera (T32, Fluke), when the laser irradiated. Tumor volumes for mice were collected every three days for 4 weeks. At the 24th day, mice were euthanatized and the organs were harvested for further study.

### 2.8. Statistics analysis

GraphPad Prism 8 software was employed for statistical analysis in this study. Multiple comparison analysis was tested by ANOVA. The results were represented as mean with SD, and p value below .05 was considered with statistical significant.

## 3. Results and discussions

### 3.1. Synthesis and characterization of obtained BMDI hybrid nanostructures

**Figure 1.**
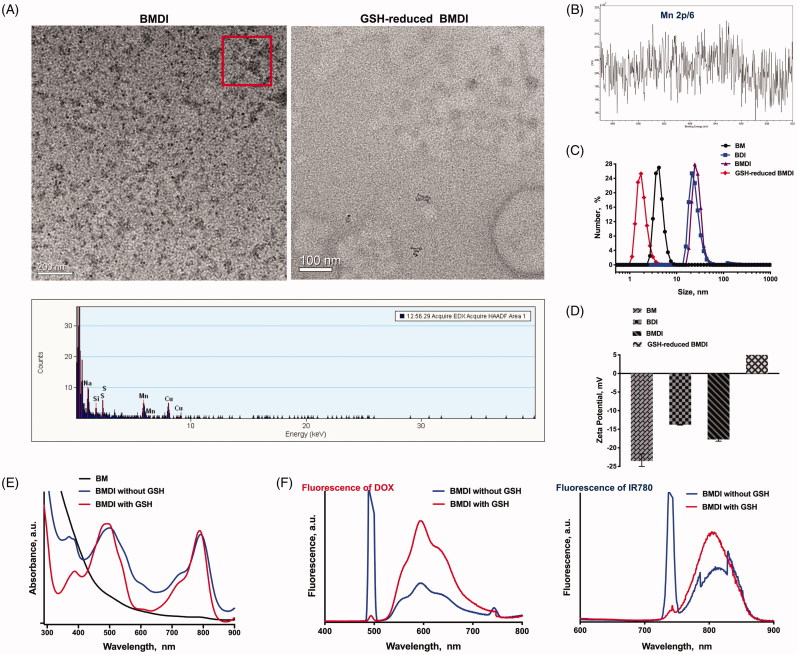
Characterization of BSA hybrid nanostructures. (A) TEM imaging of BMDI and GSH-reduced BMDI, and the EDX analysis of BMDI, the scale bar represents 100 nm; (B) X-ray photoelectron spectroscopy (XPS) analysis of Mn in BMDI nanostructures; (C) Particle size distribution and (D) Zeta-potential of BM, BDI, BMDI and GSH-reduced BMDI; (E) UV-vis spectra and (F) Fluorescence spectra of BMDI and GSH-reduced BMDI.

One-pot procedure was used to fabricate BMDI hybrid nanostructures. The isoelectronic point of BSA is of 4.7. In alkaline environment, BSA is exhibiting negative charge, which favors the attraction of metal cation. Manganese dioxide can be formed in alkaline condition (pH = 11). Therefore, we used BSA to attract Mn^2+^ in pH = 11, and then to stabilize the *in situ* grown manganese dioxide (MnO_2_). Meanwhile, DOX transfers from hydrophilic form to hydrophobic form, which can be loaded by BSA together with IR780, thus, to obtain DOX/IR780/MnO_2_ incorporated BSA (BMDI) hybrid nanostructures. The obtained BMDI nanostructures have relatively uniform particle morphology by TEM. The particle size of BMDI nanostructures is of ∼30 nm, with the ultrasmall MnO_2_ incorporation (3-4 nm, the black dot-like particles in the TEM image), and the EDX analysis further verified the composition of elements (Figure 1(A)). Furthermore, the binding energy of Mn at ∼654 and ∼641 eV in the XPS further confirmed the successful synthesis of MnO_2_ in BMDI, although the baseline of the XPS results was uneven which may be ascribed to the relative low content of MnO_2_ (Figure 1(B)). Meanwhile, the BMDI with small size distribution is well-dispersed in PBS, DMEM medium for cell culturing, and in serum. It still has a negative zeta-potential in pH = 7.4 due to the isoelectronic point of BSA is much lower than 7.4, similar with those of MnO_2_-BSA nanostructure (BM) and DOX/IR780-BSA nanostructure (BDI) (Figure 1(D)). And from the UV-vis spectrum, we also confirmed the existence of DOX and IR780 in the BMDI nanostructure (Figure 1(E)). The total content of MnO_2_ in BMDI nanostructure was detected at about 2% (m/m) by ICP-MS, and the drug-loading capacity of IR780 and DOX were calculated at 1.04 ± 0.07% and 2.12 ± 0.11%, respectively.

### 3.2. GSH-responsive property of BMDI nanostructure

Besides, some reports uncovered the activity of MnO_2_ with GSH (Cai et al., 2015). It can be used to construct GSH response nanosystem for drug controlled delivery. After dispersed in the medium with the presence of GSH (final concentration of 10 mM), no MnO_2_ ultrasmall nanoparticle was observed from the TEM image (Figure 1(A)). And the particle size measured by DLS is much small, which reduce to ∼3 nm, indicating the reaction of MnO_2_ with GSH (Figure 1(C)). On the other hand, the reversed zeta-potential of GSH-reduced BMDI could also contribute to the released of DOX and IR780 after the nanostructure decomposition of BMDI (Figure 1(D)). Moreover, dramatically enhanced fluorescence signal of DOX were further proved the dissociation of the BMDI nanostructure after treated by GSH. The intensity of the fluorescence signal of IR780 has also changed, but it is not as significant as DOX (Figure 1(F)). The results have demonstrated the successfully preparation of MnO_2_/DOX/IR780-incorporated BSA nanostructures.

**Figure 2.**
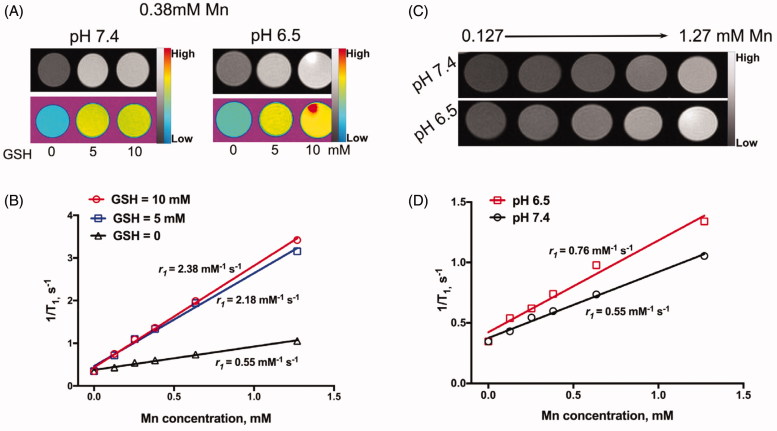
The *in vitro* environment-responsive MR imaging. (A) *In vitro* T_1_ weighted MR images of BMDI at different GSH concentrations and pH values; (B) the T_1_-weighted relaxivity (r_1_) calculated by the results of (A); (C) *In vitro* T_1_ weighted MR images of BMDI at different pH values; (D) the T_1_-weighted relaxivity (r_1_) calculated by the results of (C).

Furthermore, the dissociation of GSH-treated BMDI may release Mn^2+^, which can enhance the T_1_-weighted relaxivity in MR imaging. After treated with GSH in pH = 7.4, the r_1_ of BMDI is increased from 0.55 to 2.38 mM^−1^ s^−1^, no significant difference was observed between the BMDI treated with 5 mM GSH and that with 10 mM GSH (2.18 and 2.38 mM^−1^ s^−1^, respectively) (Figure 2(A,B)). A little increase was detected while the pH of the medium decrease (from 7.4 to 6.0) (Figure 2(C,D)). It demonstrates that the BMDI nanostructure is GSH responsive, which favor the accelerated release of Mn^2+^, making it a promising contrast agent for GSH responsive MRI.

### 3.3. Oxygen generation and responsive drug release

**Figure 3.**
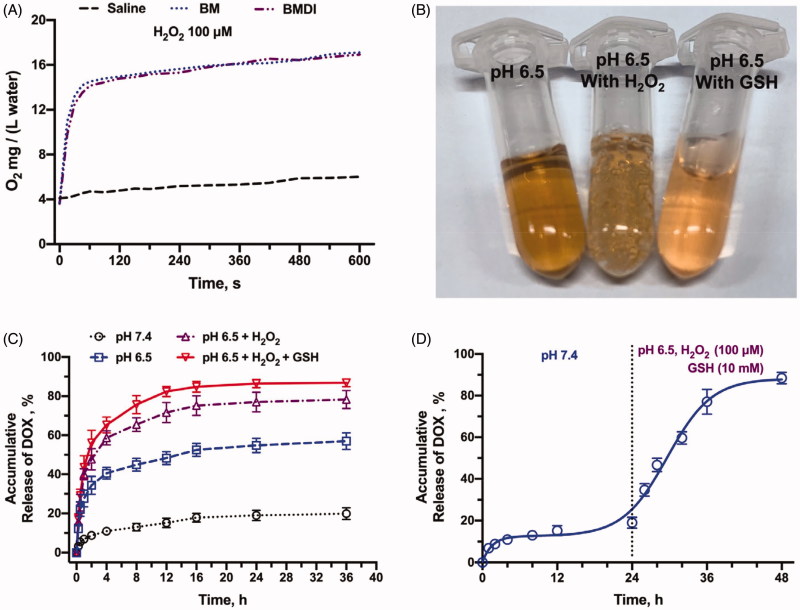
*In vitro* drug release profiles. (A) Oxygen production of BMDI and BM Nanostructure dispersed in buffer with the presence of H_2_O_2_; (B) Oxygen bubbles generation and the color alteration of BMDI dispersion with H_2_O_2_ or GSH presented at pH 6.5; (C) Accumulative DOX release of BMDI within different environments; (D) Release behavior of BMDI in alternative release buffers.

Moreover, the MnO_2_ can also react with H_2_O_2_ to generate oxygen (Chen et al., 2016). While the BMDI nanostructure was dispersed in the deoxygenated medium containing H_2_O_2_, oxygen generation was detected (the O_2_ in water increased from ∼3.9 to ∼14 mg/(L water) in the very first 45 seconds, and became saturation in 1 min) (Figure 3(A)). Numerous bubbles were obviously observed (Figure 3(B)). What’s interesting is few oxygen generation was detected while the BMDI was dispersed in the deoxygenated medium containing GSH. It may be ascribed to the different reacting mechanism while BMDI reacted with GSH.

The drug release behavior of BMDI in different buffers was further investigated according to the previous study. In pH 7.4, the DOX was released relatively slow (<20% in 24 h). While the pH dropped to 6.5, accelerated drug release was detected (more than 50% in 24 h). The existence of either H_2_O_2_ or H_2_O_2_ combined with GSH further accelerated the release of DOX from BMDI (>70% and >82% of DOX were released, respectively) (Figure 3(C)). The H_2_O_2_/GSH accelerated drug release behavior of BMDI was more obvious in Figure 3(D). Dramatically drug release was detected after the buffer was changed from pH 7.4 buffer to pH 7.4 with the addition of H_2_O_2_ and GSH (100uM and 10 mM, respectively). The results have revealed that the BMDI is H_2_O_2_/GSH responsive, which favor the realization of stimuli-triggered drug release to reduce adverse effects caused by uncontrollable drug release (Xu et al., 2018).

### 3.4. Cellular uptake and cytotoxicity

**Figure 4.**
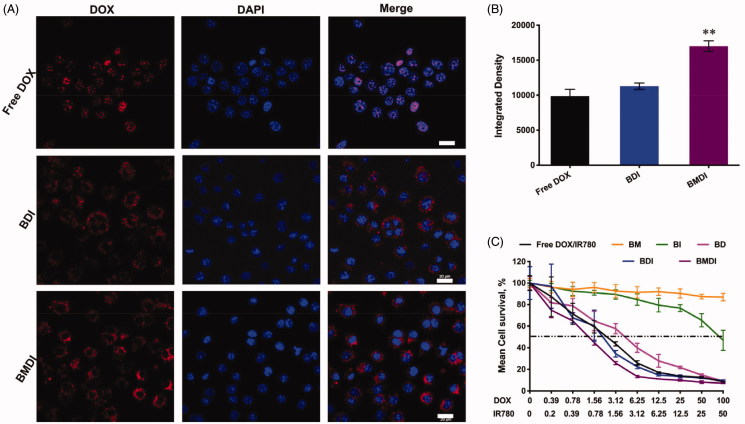
(A) Cell uptake images with different formulations after 4 h incubations. The red fluorescence represents DOX, and the nucleus were labeled with blue fluorescence (*n* = 3, ‘**’ *p* < .01 was considered with significance. Scale bar is 20 μm); (B) Semi-quantitative analysis based on (A); (C) Cell survival after treating with free IR780 and DOX, BM, BI, BD, BDI or BMDI nanostructure.

We then evaluated the anticancer performance of BMDI *in vitro*. Before to assess the cytotoxicity of BMDI to cancer cells, we first investigated the cellular uptake of cancer cells to BMDI. More DOX (red fluorescence) was detected after it incorporated with IR780/MnO_2_/BSA. Different with free DOX which fast localized in the nucleus, the BDI was mainly localized in the cytoplasm after engulfed by cancer cells. Similar result was obtained in the case of BMDI, which suggested the decomposition of the MnO_2_ enquired the localization in cytoplasm (Figure 4(A)). Cellular uptake level was calculated by ImageJ in the revised manuscript. The results revealed that the higher uptake proportion for BMDI-treated cancer cells, compared to the BDI- or free DOX-treated cells. BSA-based nanostructure mediated protein interaction with the cell membrane could further promote the cellular uptake behavior of the cancer cell to DOX (Figure 4(B)).

By MTT assay, we have evaluated the cytotoxicity of BMDI to cancer cells. The IC50 of free DOX/IR780 mixture to cancer cells was ∼2.7 ug/mL (DOX), which is similar with BD nanostructure. After the introduction of MnO_2_, the IC50 of BMDI reduced to 0.98 ug/mL (DOX), while at these concentration, BM (with MnO_2_ of 1 ug/mL) did not exhibit any cytotoxicity to cancer cells (Figure 4(C)). It indicates that the MnO_2_ or its degradation enhanced the cytotoxicity of DOX. Meanwhile, the BSA absorbed IR780 (BI) present alleviated cytotoxity.

**Figure 5.**
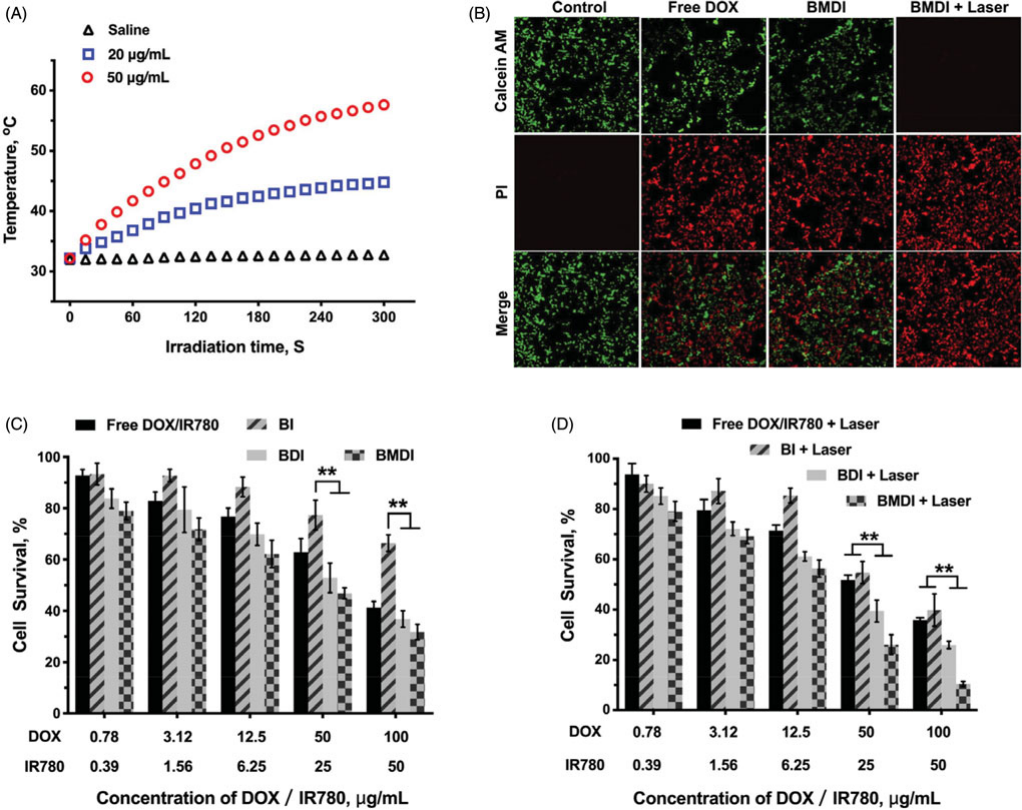
(A) Temperature curves of BMDI dispersions with different concentrations exposed to NIR laser irradiation; (B) Living/dead cell-staining after BMDI treatments with or without photothermal ablation; Cell survival assay for different BSA-based formulations treatment: for (C) without laser irradiation, and for (D) with laser irradiation. Data reported represent mean ± SD (*n* = 3, and ‘*’ *p* < .05).

Furthermore, because the IR780 can be served as photosensitizer to generate heat which can be used to induce the necrosis of cancer cell directly, we further evaluated the anticancer performance of BMDI-mediated photothermal therapy *in vitro* (Yang et al., 2019b). Excellent photothermal conversion of BMDI was observed (the temperature of the BMDI aqueous dispersions (20 μg/mL and 50 μg/mL) raised from 32 °C to 45 °C and 58 °C, respectively) (Figure 5(A)). And the growth of the cancer cells treated with BMDI-mediated PTT was also efficiently inhibited. Similar cancer cell death was observed between the free DOX treated group and the BMDI-treated group in live/death assay. After the introduction of PTT, almost all the growth of cancer cells was inhibited (Figure 5(B)). Notably, BMDI nanostructure presented superior inhibition on tumor cell growth, compared to the free DOX/IR780 or BI or BDI did in dark, implying that oxygen generation by MnO_2_ could improve the chemotherapeutic efficacy. More importantly, the combined photo-chemotherapy of BMDI showed significant therapeutic efficacy. While the free DOX/IR780, BI and BDI plus laser treatments still present incomplete ablation of cancer cells, which would ascribe to their relative lower uptake efficiency (Figure 5(C,D)).

### 3.5. Tumor targeting *in vivo*

**Figure 6.**
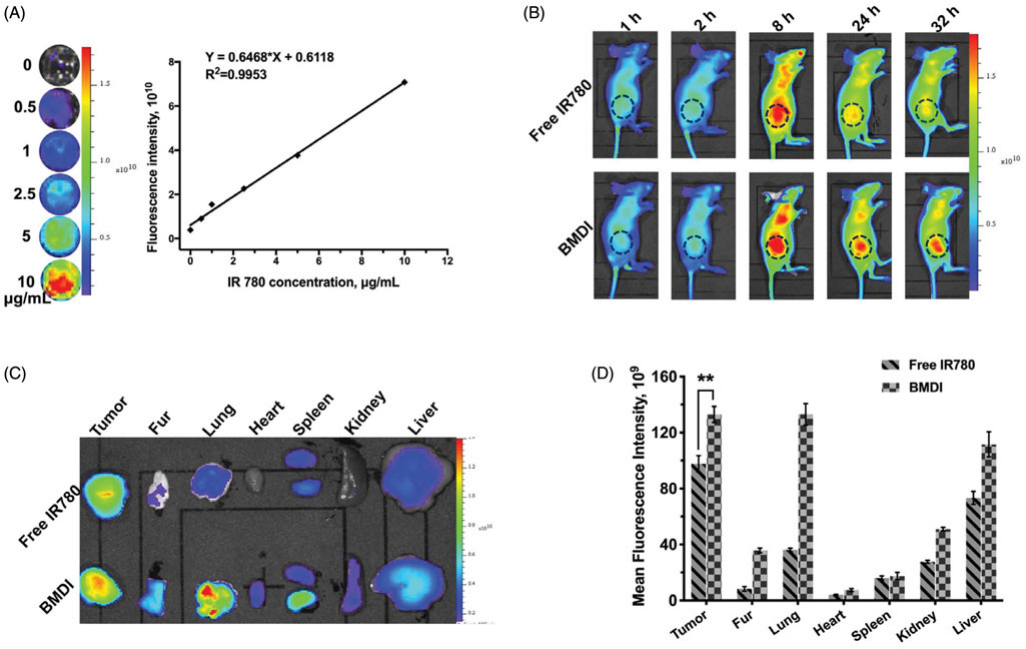
Biodistribution of BMDI *in vivo*. (A) The linearity of fluorescence intensity of BMDI dispersion *in vitro*; (B) Living fluorescence imaging of the nanostructures distribution on MCF-7 tumor-bearing mice; (C) *Ex vivo* fluorescence imaging of tumor, fur and organs eviscerated from mice at the end of the imaging experiment; (D) Semi-quantification of fluorescent intensity based on the results of (C) (‘**’ *p* < .01).

Tumor targeting is an important property of nanomedicines (Xiao et al., 2018). The tumor targeting performance of BMDI is evaluated *in vivo*. We first identified the relationship between the fluorescence intensity of BMDI and its concentration, to figure out the concentration range of BMDI while the fluorescence intensity is linearly increased as the increase of BMDI concentration (Figure 6(A)). We found that the fluorescence intensity of BMDI is linearly increased while the BMDI concentration is increased from 0 to 10 ug/mL. Then, mice with MCF-7 breast tumor burden were utilized for tumor targeting investigation *in vivo*. After intravenously injected, strong fluorescence signal was detected in the tumor region, and the intensity of BMDI treated group is much stronger than the free IR780 treated group in 24 h after injection of BMDI or free IR780 (Figure 6(B)). The fluorescence intensities of the organs eviscerated in 48 h after the injection further indicated the enhanced accumulation and retention of IR780 in cancer tissues (Figure 6(C,D)). It demonstrated the incorporation of MnO_2_ and DOX/IR780 with BSA indeed enhanced the tumor-targeting of therapeutics.

### 3.6. Anticancer performance *in vivo*

**Figure 7.**
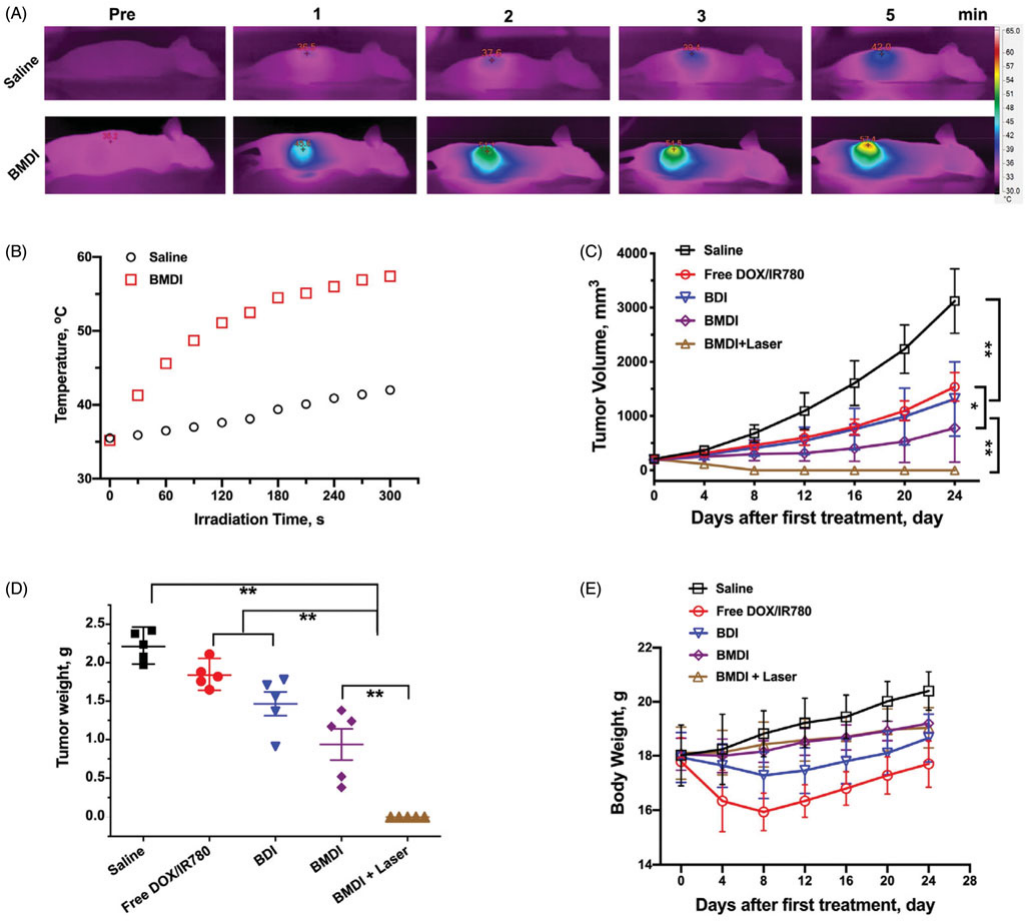
The antitumor investigation of BMDI. (A) *In vivo* photothermal conversion of BMDI; (B) Relationships of photothermal conversion *in vivo*; (C) Tumor volume variation of groups with various administrations; (D) Tumor weight from each group of the mice; (E) Mean body weights of the mice for each treatment. (*n* = 5, means ± SD, ‘**’ represents *p* < .01).

Based on the above results of cytotoxicity in vitro and tumor targeting in vivo, we further evaluated the tumor growth inhibition of BMDI in vivo as well as the combination of BMDI mediated chemotherapy and photothermal therapy. The photothermal conversion of BMDI *in vivo* was assessed initially (Figure 7(A,B)). After irradiated for 1 min, the temperature of the tumor region treated with BMDI was increased from ∼35 °C to 45 °C, which is higher than the temperature used for effective thermal therapy (while the temperature is higher than 43 °C, the proliferation of cancer cells was effectively inhibited). And the temperature was further increased to ∼57 °C while the irradiation time was prolonged to 5 min. The temperature of the tumor treated with saline was just increased from ∼35 °C to ∼42 °C. It indicated that the BMDI still has effective photothermal conversion in vivo, which can be acted as agent for PTT.

Then, we evaluated the anticancer performance of BMDI *in vivo*. Similar (no significant difference) tumor growth inhibitions were observed between the free DOX/IR780 treated mice and the BDI treated mice, while the tumor growth inhibiting rates were at ∼50%. In the BMDI treated group, the tumor growth inhibiting rate was increased to ∼70%. With the introduction of PTT, the tumor growth inhibiting rate was further increased to ∼100% (Figure 7(C,D)). The results indicated that the DOX has relative strong anticancer activity. In the case of BMDI, the introduction of MnO_2_ could alleviate the hypoxia condition of the tumor, then change the metabolic environment of the tumor, thus, enhance the therapeutic outcome of DOX mediated chemotherapy. And the introduction of PTT can further induce necrosis of cancer cells and enhance the activity of chemotherapeutics, resulting a highest tumor growth inhibiting rate (Figure 7(D)). Additionally, the serious adverse effects of free DOX/IR780 and BDI formulation caused obvious emaciation for mice, while the body weight of mice with BMDI and BMDI plus laser treated increased slightly, indicating the biosafety of BMDI nanostructure (Figure 7(E)).

**Figure 8.**
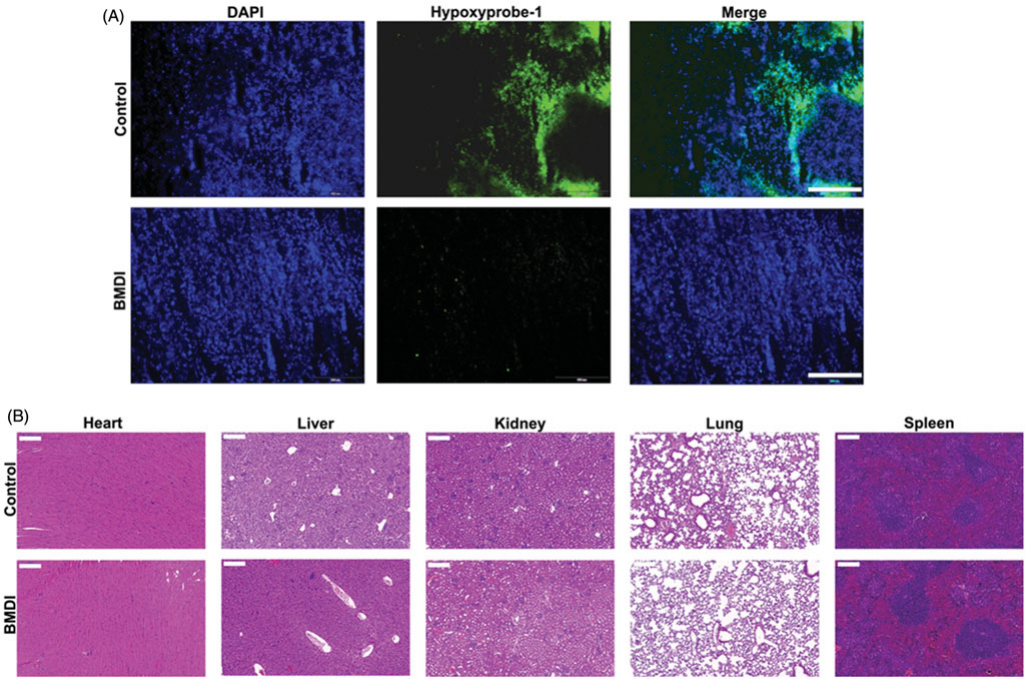
(A) Immunofluorescence images of the tumor slides with hypoxia biomarker staining for control and BMDI-treated groups; (B) Histochemical images of major organs (heart, liver, kidney, lung and spleen) for control and BMDI-treated groups. (Scale bar is 200 µm).

Amount of research work have been reported that the tumor microenvironment (TME) regulation, especially the alleviation of tumor hypoxia might reverse the suppression of the activity of chemotherapeutics (Song et al., 2016; Zhu et al., 2018). To confirmed the therapeutic effect of self-oxygen generation nanomaterials, the tumor slides were prepared and stained with a Hypoxyprobe-1 kit after the 3 days of treatment. The hypoxia situation of tumor in the BMDI-treated group was dramatically alleviated, compared to that of control group (Figure 8(A)). It demonstrates that the introduction of MnO_2_ indeed improve the therapeutic activity of chemodrug by remodeling the TME. Furthermore, no obvious histological alterations were observed in the H&E-staining of major organs slides for the BMDI-treated group, also indicating the biocompatibility of the BMDI nanostructure (Figure 8(B)). And it also can be served as an effective candidate for combination of phototherapy and chemotherapy.

## 4. Conclusion

In summary, we have successfully prepared a hybrid nanostructure by in situ precipitation of manganese dioxide in bovine serum albumin (BSA) with the incorporation of doxorubicin (DOX) and IR780. The obtained hybrid nanostructure (BMDI) is H_2_O_2_/GSH dual responsive. It can react with H_2_O_2_ to generate oxygen, thus, alleviate tumor hypoxia. And the H_2_O_2_/GSH responsive also favor the realization of stimuli-triggered drug release. As the result of stimulated MnO_2_ degradation, the released Mn ions further enhanced the magnetic resonance signal which made the BMDI a promising contrast agent for MRI. Moreover, the introduction of MnO_2_ has enhanced the anticancer activity of DOX *in vitro* and *in vivo*, and efficiently suppressed the tumor growth. By further introducing with photothermal therapy (PTT), the tumor growth was almostly inhibited. It suggested that the BMDI hybrid nanostructure could be a promising candidate for tumor growth inhibition as therapeutics carrier.
